# Robustness quantification of a mutant library screen revealed key genetic markers in yeast

**DOI:** 10.1186/s12934-024-02490-2

**Published:** 2024-08-04

**Authors:** Cecilia Trivellin, Luca Torello Pianale, Lisbeth Olsson

**Affiliations:** Department of Life Sciences, Division of Industrial Biotechnology, Chalmers University of Technology, 412 96 Gothenburg, Sweden

**Keywords:** Perturbations, Bioprocesses, MET28, High-throughput, Microbial robustness

## Abstract

**Background:**

Microbial robustness is crucial for developing cell factories that maintain consistent performance in a challenging environment such as large-scale bioreactors. Although tools exist to assess and understand robustness at a phenotypic level, the underlying metabolic and genetic mechanisms are not well defined, which limits our ability to engineer more strains with robust functions.

**Results:**

This study encompassed four steps. (I) Fitness and robustness were analyzed from a published dataset of yeast mutants grown in multiple environments. (II) Genes and metabolic processes affecting robustness or fitness were identified, and 14 of these genes were deleted in *Saccharomyces cerevisiae* CEN.PK113-7D. (III) The mutants bearing gene deletions were cultivated in three perturbation spaces mimicking typical industrial processes. (IV) Fitness and robustness were determined for each mutant in each perturbation space. We report that robustness varied according to the perturbation space. We identified genes associated with increased robustness such as MET28, linked to sulfur metabolism; as well as genes associated with decreased robustness, including TIR3 and WWM1, both involved in stress response and apoptosis.

**Conclusion:**

The present study demonstrates how phenomics datasets can be analyzed to reveal the relationship between phenotypic response and associated genes. Specifically, robustness analysis makes it possible to study the influence of single genes and metabolic processes on stable microbial performance in different perturbation spaces. Ultimately, this information can be used to enhance robustness in targeted strains.

**Supplementary Information:**

The online version contains supplementary material available at 10.1186/s12934-024-02490-2.

## Background

Robustness denotes the ability of a system to maintain a stable performance even in the face of internal or external challenges [51, 60, 64]. In biology, robustness is generally referred to a specific phenotype, such as ethanol productivity in yeast or the maximum specific growth rate. However, it can be considered also as a holistic property, whereby the stability of various phenotypes is considered simultaneously under multiple perturbations [70]. It should be noted that robustness is different from fitness (i.e., performance), which refers to the specific value of a phenotype in a given environment. Robustness can be defined in relation to the mean performance across a range of perturbations [90, 99] or as a performance ratio when a perturbation is applied compared to a control condition [52]. Given that perturbations and environments vary depending on the context (e.g., a substrate containing organic acids or an environment with elevated temperature), selecting a specific control condition becomes an arbitrary choice. Therefore, calculating robustness as the mean phenotype across perturbations [39, 99] has broader applicability compared to determining robustness by referencing the phenotypic state in a control condition [13, 52].

Previously, we described a robustness quantification method based on the Fano factor, which is free from arbitrary controls, frequency-independent, and dimensionless [90]. It can be applied to phenotypic values collected in a perturbation space (a set of relevant conditions) for one or multiple systems (such as microorganisms). A significant advantage of employing this method is its compatibility with existing datasets containing phenotypic information. Over the last 30 years, advances in screening technologies and data analysis have enabled high-throughput phenotyping and phenomics mapping [65, 95, 100]. Phenomics datasets are an important tool for unravelling complex biological patterns. For example, extensive phenotypic datasets can be used to understand genotype-to-phenotype maps [71], gene expression patterns under different conditions [40] or correlations among distinct traits [75]. Gene deletion libraries are instrumental in assessing the role of genes in specific cellular processes by observing phenotypic changes [5, 81, 86]. Here, we show for the first time how fitness data from a yeast deletion collection screen [22] can be used as input for microbial robustness quantification across different environments to identify genetic and metabolic markers of robustness.

Phenotypic stability under different conditions in various perturbation spaces (e.g., lignocellulose hydrolysate fermentation) points to strains with robust phenotypes. However, unravelling the mechanism responsible for the robustness of specific traits is challenging [38, 63] and limits the ability to perform rational strain engineering. The primary difficulty in understanding robustness mechanisms lies in linking phenotypes with genetic architecture, regulatory networks, and post-translational modifications [47, 67, 78]. While the role of single/multiple genes in mediating tolerance towards specific perturbations has been explored [8, 46, 81], they have not been investigated as overall robustness markers. Exploring the role of single genes will improve our understanding of robustness mechanisms.

The overall aim of the present study was to identify genetic markers of microbial robustness. First, we performed robustness analysis on a reference dataset [22] containing fitness data (i.e., colony size) in 14 conditions for more than 4000 *Saccharomyces cerevisiae* mutants (BY4741 or Y7092 background) carrying non-essential gene deletions as well as temperature-sensitive essential alleles. Second, we identified mutants with the best and worst robustness using percentile scores and replicated 14 of the corresponding non-essential gene deletions in the laboratory CEN.PK113-7D strain. To test the phenotype-perturbation space specificity of robustness [39, 64], the 14 single-gene mutants, the parental CEN.PK113-7D strain, and the Ethanol Red industrial strain were cultivated in three different perturbation spaces. Finally, we calculated the robustness of the 14 mutants to reveal genetic targets that could enhance their stable performance. Application of the same strategy to larger libraries and more perturbation spaces will boost our understanding of the mechanisms underlying robustness.

## Results

**Fig. 1:**
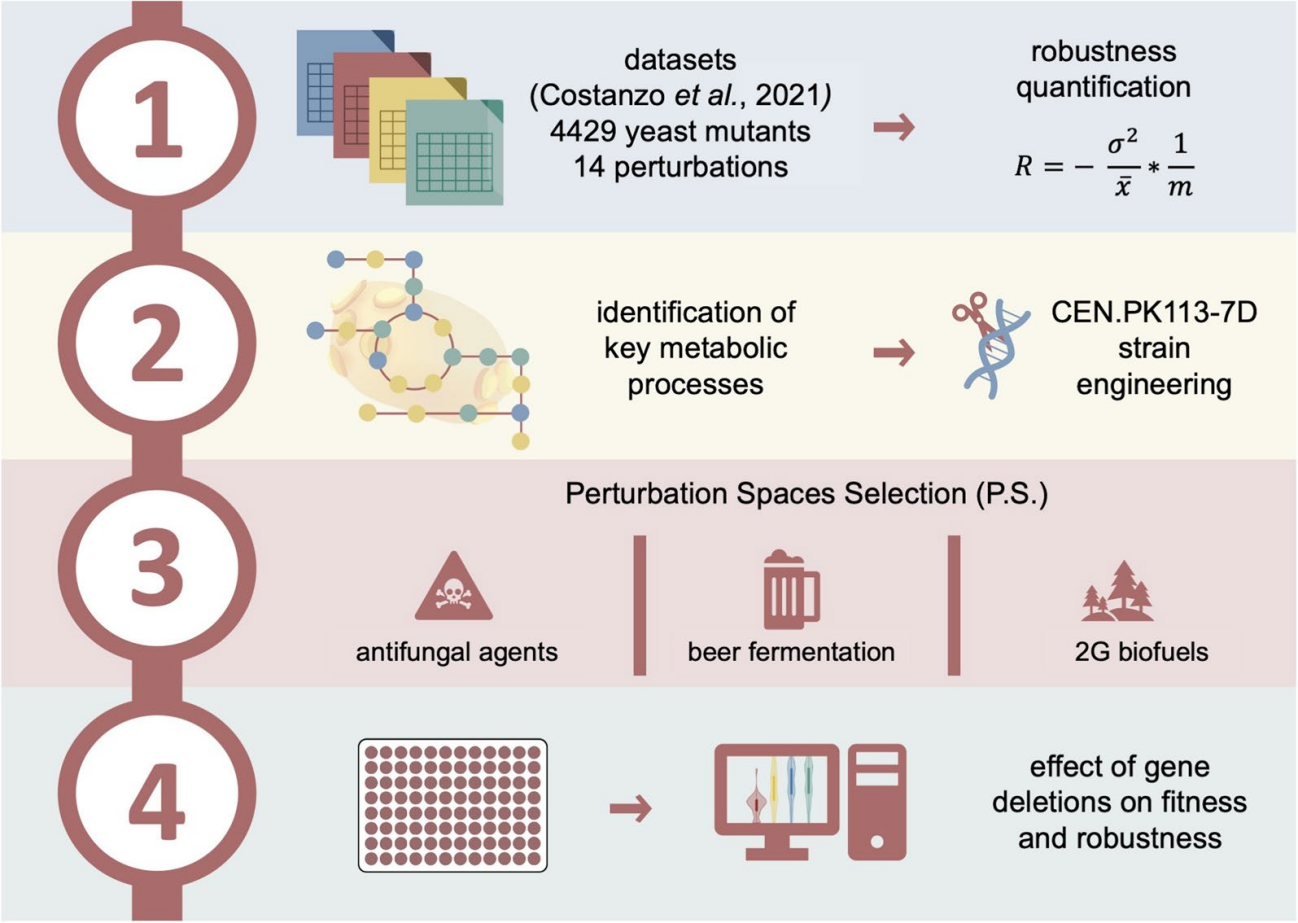
Workflow of the study divided into four steps. 1. Calculation of robustness from the reference dataset. 2. Identification of genes and metabolic processes related to high/low fitness or robustness, followed by their transfer to *S. cerevisiae* CEN.PK113-7D for further characterization. 3. Cultivation of mutants bearing gene deletions in three perturbation spaces each composed of 16 single conditions. 4. Calculation of fitness and robustness for each strain and perturbation space

The workflow in the present study encompassed four main steps (Fig. 1). In the first two steps, fitness data for over 4000 mutants with non-essential gene deletions and temperature-sensitive alleles of essential genes [22] were employed to quantify robustness using our previously published method [90]. Mutants were ranked based on their fitness and robustness scores, and specific non-essential genes from the top and bottom ten strains were chosen (Material and Methods). In steps 3 and 4, the 14 single-gene deletions were introduced into *S. cerevisiae* CEN.PK113-7D, a strain with favorable growth characteristics under industrially relevant conditions and ease of manipulation [36, 68]. Mutants carrying the gene deletions plus the control and Ethanol Red were cultivated in three distinct perturbation spaces (second-generation biomass fermentation, beer fermentation, and conditions from the reference dataset) to investigate the impact of non-essential gene deletions on fitness and robustness.

### Robustness analysis of fitness data identifies relevant genes and metabolic processes

Robustness was calculated from a dataset containing fitness data of 4429 mutants (BY4741 or Y7092) bearing deletions in non-essential genes, as well as temperature-sensitive alleles corresponding to 553 essential genes [10, 22, 23]. In the reference dataset, mutants were grown under 14 conditions (combination of sugars and antifungal agents) and fitness was reported as normalized colony size [10]. Robustness analysis using the reference dataset identified 67 mutants with maximal robustness (R = 0). Of these, seven carried mutations in temperature-sensitive alleles and 60 carried non-essential gene deletions.

**Fig. 2:**
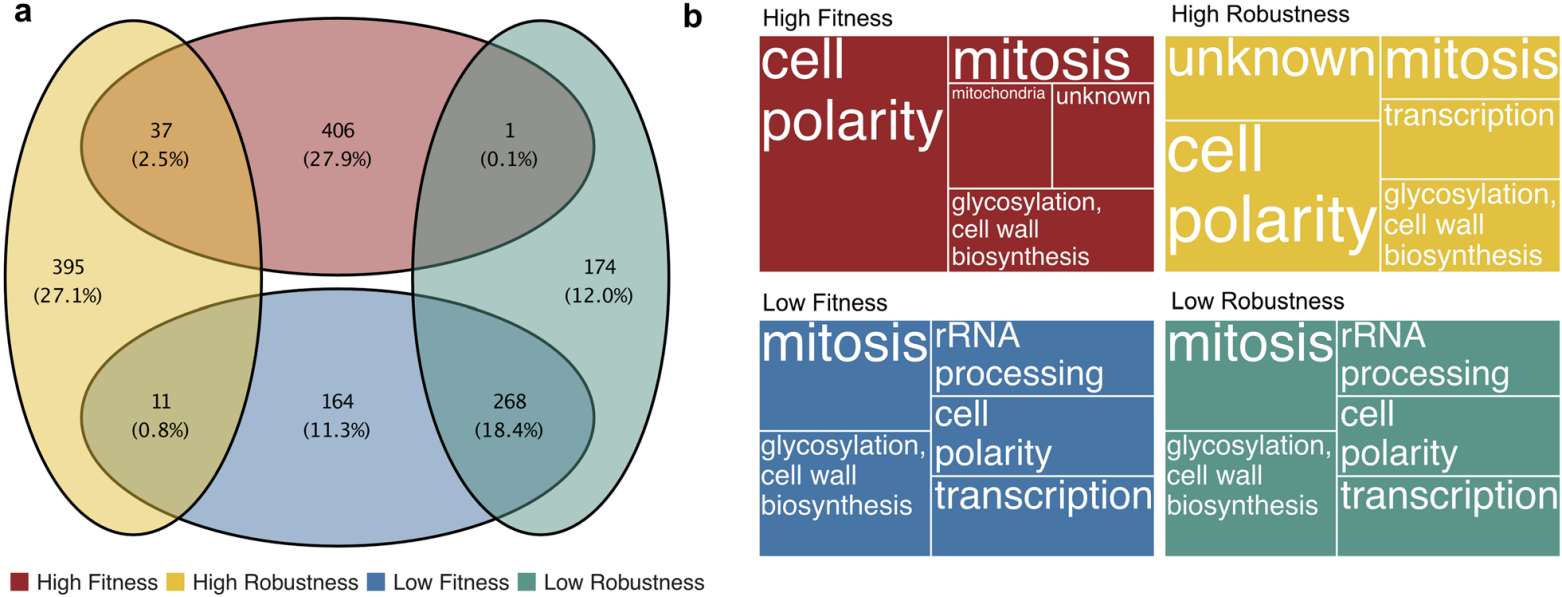
Overlap of mutants and metabolic processes in the reference dataset. **a** Venn diagram presenting logical connections among the four data sets (10th and 90th percentiles) representing mutants with the highest and lowest values of fitness and robustness. For each set, the number of mutants and overall percentage of mutants found in the respective categories are reported. **b** Treemap illustrating the top five SAFE network regions most prevalent in the mutants exhibiting the highest and lowest values of robustness and fitness. Larger squares indicate a higher number of strains associated with each specific metabolic process

To examine the link between fitness and robustness, we selected the top and bottom mutants (90th and 10th percentiles, respectively) from our dataset (distribution of fitness and robustness are shown in Figure S1). The four resulting datasets represented approximately one-eighth of all mutants. Percentiles were chosen to achieve a balance between statistical significance and the ability to effectively showcase strains with the desired characteristics (Fig. 2a). Mutants with either high fitness or high robustness were the most prevalent (27.9% and 27.1% respectively), while those exhibiting high values for both parameters were much less abundant (2.5%). A good percentage of mutants (18.4%) exhibited both low fitness and low robustness. Finally, mutants with either low robustness or low fitness amounted to 12.0% and 11.3% of the total, respectively. The divergence between fitness and robustness, whereby strains exhibit a high value of one parameter but a low value of the other could suggests a trade-off [50]. However, only 0.1% of mutants displayed high fitness and low robustness, while 0.8% did the opposite, indicating that trade-offs were uncommon in this analyzed context.

The global genetic profile similarity network was previously annotated for yeast using spatial analysis of functional enrichment (SAFE). This method allowed the identification of metabolic processes linked to most non-essential and essential genes [9, 22]. After extracting mutants according to either the 10th or 90th percentiles of robustness and fitness, the ID of each mutant was merged with the SAFE network region (Table S1). Most genes with elevated robustness were associated with “cell polarity”, “mitosis” or “unknown” regions (Fig. 2b). The same processes came up also when considering strains with high fitness, although fewer fell into the “unknown” regions category. Overlapping SAFE regions appeared in the low robustness and low fitness sets in almost the same proportion, and with “rRNA processing” emerging exclusively in these sets. Instead, “Mitochondria” appeared only in the high fitness set. When counting mutants in each SAFE region, categories such as cell polarity, glycosylation, mitochondria, mitosis, and "unknown" were notably the most frequent. As a result, any subset of data containing these regions might appear to have inflated numbers simply due to their higher prevalence in the reference dataset. To assess whether the fitness and robustness of each SAFE region deviated significantly from that of the overall distribution including all SAFE regions, we employed a Wilcoxon test (Figure S2). We found that several SAFE regions, such as "unknown" and "cell polarity," significantly differed from the background population in both fitness and robustness. Additionally, we observed that "peroxisome" and "transcription" were significantly different only in robustness compared to the reference dataset, while "glycosylation and cell wall biosynthesis" showed significance only in fitness.

### Experimental setup for the identification of robustness genetic markers

Among the mutants with either the highest or lowest robustness (10th and 90th percentiles) in the reference dataset, a total of 14 genes (Table 1) were individually deleted from *S. cerevisiae* CEN.PK113-7D using CRISPR-Cas9 genome editing technology. Three genes were picked among the ten with the highest robustness (different from zero) in the reference dataset: QDR1, MET28, and MRP31 (group HR). Six genes were picked among the ten with robustness equal to zero and the highest fitness: HCM1, GBP2, RPS14A, RPS14B, OCA4, and MSH3 (group R0HD). Finally, five genes were chosen among the ten genes with the lowest robustness in the reference dataset: BCH1, WWM1, HLJ1, TIR3, and SMA2 (group LR). The 14 selected genes represented a wide range of metabolic activities and cell processes, based on the SAFE regions reported in the reference dataset (Table 1). The parental CEN.PK113-7D strain and Ethanol Red, an industrial strain commonly used in first-generation ethanol production, were included as controls [48].

**Table 1:** List of deleted genes in the CEN.PK113-7D strain

Gene	SAFE network region	Description
*QDR1*^a^	NA	Multidrug transporter
*MET28*^a^	Transcription	Regulation of sulfur metabolism
*MRP13*^a^	Mitochondria	Mitochondrial ribosomal protein
*HCM1*^b^	Mitosis	Forkhead transcription factor, suppressor of calmodulin
*GBP2*^b^	Nuclear transport	RNA-binding protein involved in translation repression
*RPS14a*^b^	rRNA processing	Ribosomal protein of the small subunit
*RPS14b*^b^	rRNA processing	Ribosomal protein of the small subunit
*OCA4*^b^	Glycosylation, cell wall biosynthesis	Oxidant-induced cell-cycle arrest
*MSH3*^b^	DNA replication/repair	Mismatch repair protein
*BCH1*^c^	Peroxisome	Member of the chaps family
*WWM1*^c^	Cell polarity	Protein interacting with metacaspase
*HLJ1*^c^	Protein turnover	Co-chaperone for Hsp40p
*TIR3*^c^	Mitosis	Cell wall mannoprotein
*SMA2*^c^	DNA replication/repair	Spore membrane assembly

^a^Highest R (HR)

^b^R = 0 and highest fitness (R0HF)

^c^Lowest R (LR)

The 14 deletion mutants were cultivated in three distinct perturbation spaces. The first perturbation space included a set of conditions similar to those tested in the reference dataset [22], which encompassed mainly antifungal compounds and sugars dissolved in chemically defined medium. The second perturbation space included different combinations of malts, hops, aromas, and fining agents used in beer fermentation. The third perturbation space included conditions relevant to the fermentation of lignocellulose hydrolysates, namely acids, phenolics, aldehydes, salts, and sugars (as outlined in Material and methods). The beer fermentation space was included because, in general, the materials used to make beer are less likely to inhibit yeast metabolism compared to lignocellulose hydrolysates. However, compounds released during heat treatment of malt, the malts used in the production of dark beer, and the melanoidins released from Maillard reactions can inhibit yeast metabolism [73]. The three perturbation spaces were intentionally designed to encompass different bioprocesses, each affecting yeast metabolism in distinct ways. This approach allowed us to calculate robustness within a broader and more varied environment.

### The Costanzo perturbation space (CPS): in silico robustness quantification of the reference dataset does not match experimental robustness

The selected 14 mutants plus controls were cultivated in the 16 perturbations similar to the ones in the reference dataset (Table 2). The perturbation set is hereafter referred to as CPS. Notably, there were three key distinctions between the present CPS and the original one (CPSo) employed by Costanzo and colleagues: (I) fitness was quantified by specific growth rate in liquid medium instead of normalized colony size, (II) all perturbations were replicated using chemically defined Delft medium instead of YEPD plates [10], and (III) only eight of the 14 antifungal agents matched those in CPSo (e.g., Benomyl), while the rest were similar (e.g., Thapsigargin in place of Tunicamycin). The set that included only the eight perturbations containing the same antifungal agent as the CPSo is hereafter referred to as CPS reduced (CPSr).

**Table 2:** List of sugars and antifungal agents (with relative concentrations and medium code) added to delft minimal medium in the CPS

Perturbation code	Carbon source	Antifungal agent	References
CPS_1	Glucose (20 g/L)	/	
CPS_2	Sucrose (20 g/L)	/	
CPS_3*	Glucose (20 g/L)	/	
CPS_4	Galactose (20 g/L)	/	
CPS_5	Sorbitol (1 M)	/	
CPS_6	Glucose (20 g/L)	Actinomycin D [50–76-0] (20 μM)	[96]
CPS_7	Glucose (20 g/L)	Benomyl [17804-35-2] (30 μg/mL)	[19, 20, 35, 54]
CPS_8	Glucose (20 g/L)	Geneticin [108321-42-2] (200 μg/mL)	[42]
CPS_9	Glucose (20 g/L)	Caspofungin diacetate [179463-17-3] (0.1 μg/mL)	[15]
CPS_10	Glucose (20 g/L)	Bafilomycin [88899-55-2] (100 nM)	[33]
CPS_11	Glucose (20 g/L)	Puromycin dihydrochloride [58–58-2] (0.1 μg/mL)	[17]
CPS_12	Glucose (20 g/L)	Fluconazole [86386-73-4] (16 μg/mL)	[1]
CPS_13	Glucose (20 g/L)	Geldanamycin [30562-34-6] (10 μM)	[85]
CPS_14	Glucose (20 g/L)	Nigericin sodium salt [28643-80-3] (50 μg/mL)	[57]
CPS_15	Glucose (20 g/L)	Rapamycin [53123-88-9] (100 nM)	[53]
CPS_16	Glucose (20 g/L)	Thapsigargin [67526-95-8] (1 mg/mL)	[82]

^a^CPS_3 did not contain delft minimal medium as a base, but was made with YPD (20 g/L Peptone, 10 g/L yeast extract, and 20 g/L glucose)

**Fig. 3:**
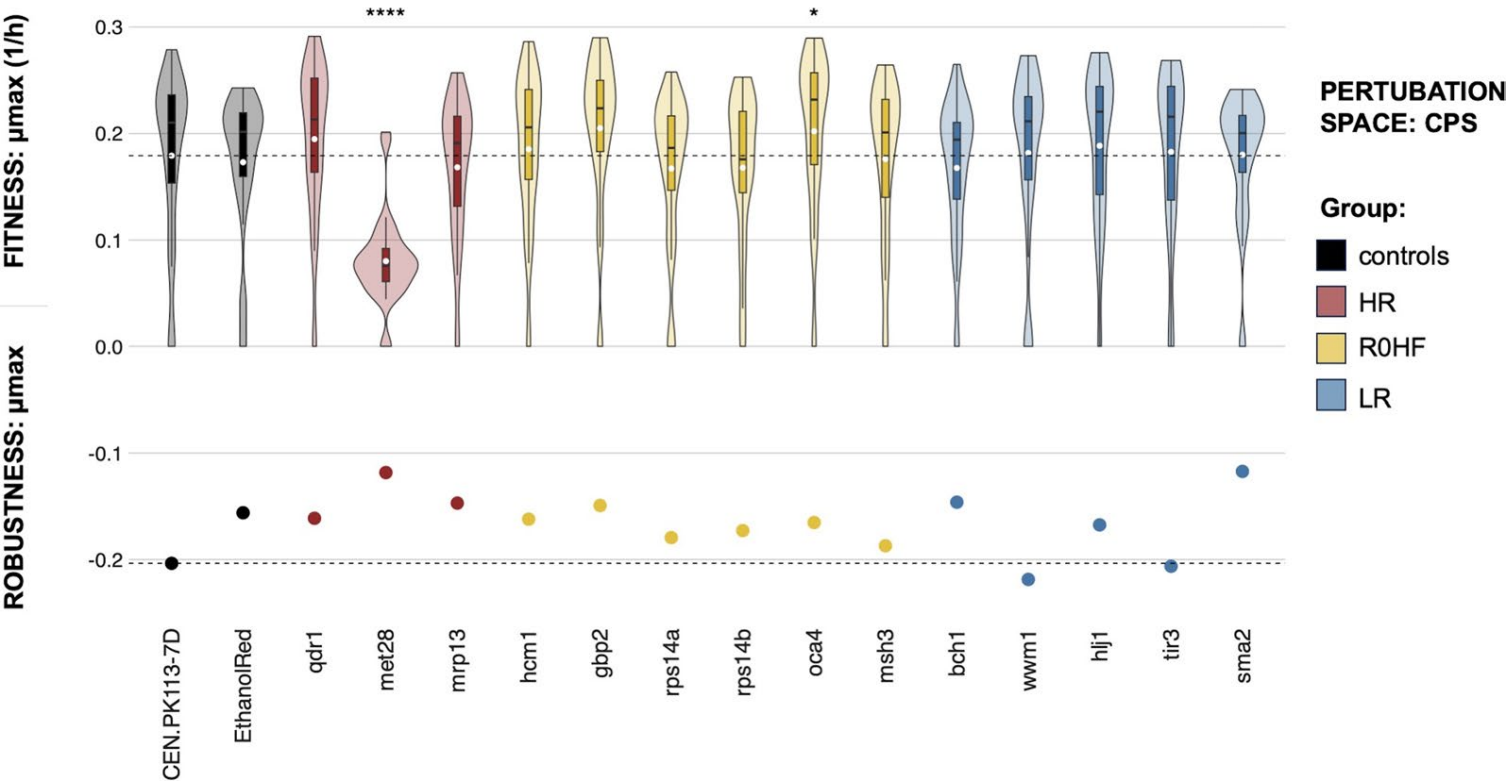
Fitness and robustness in the CPS (Delft medium + antifungal agents). The y-axis (0;0.3) represents the maximum specific growth rate (1/h) across all strains (controls and mutants with single gene deletions) and perturbations (n = 48, 16 conditions, 3 replicates), with the black line in the boxplot indicating the median of the distribution and the white dot indicating the mean. Significant difference between the parental strain and the mutants is denoted by p-values obtained from Wilcox tests (*p < 0.05, ****p < 0.0001, not significant scores are omitted). Robustness of the maximum specific growth rate is shown by dots on the y-axis (− 0.2;0), while the horizontal black line denotes robustness and fitness of the CEN.PK113-7D parental strain. Groups are colored differently based on analysis of the reference dataset (HR: highest robustness score different from zero; R0HF: robustness equal to zero and highest fitness score; LR: lowest robustness score; controls: CEN.PK113-7D parental and Ethanol Red)

Within the CPS, most mutants showed fitness distributions analogous to those of the parental strain, except for *met28* and *oca4* (Fig. 3). The *gbp2* mutant exhibited the highest average fitness, although its distribution did not differ significantly from that of the parental strain. In contrast, the *met28* mutant displayed the lowest average fitness, even though its normalized colony size in the CPSo was comparable to that of the control strain. Consequently, deletion of MET28 led to contrasting behaviors between the CPS and CPSo. This distinction persisted even in CPSr (Figure S3). The *tir3* mutant was among the ten candidates with the lowest fitness based on CPSo data; however, here, its mean specific growth rate was comparable to that of the parental strain.

Overall robustness calculated using the complete set of 16 conditions had a mean of -0.16 and a standard deviation of 0.02. Nearly all mutants showed a higher robustness than the parental strain (Fig. 3), with *sma2* exhibiting the highest value (R = − 0.12). Only *wwm1* and *tir3* displayed a reduced robustness (R = − 0.22 and R = − 0.21, respectively). In the CPSr, a distinct pattern emerged, with *met28* displaying the highest robustness (R = − 0.03) and the parental strain a very low one (R = − 0.22) (Figure S3).

A comparison of CPS and CPSr results (Fig. 3 and Figure S3) with the normalized colony size in CPSo (Figure S4) failed to reveal a distinct and consistent pattern among the groups defined by high or low fitness and robustness (Table 1, HR; R0HF; LR). Although not all mutants matched the robustness values derived from CPSo analysis, it is noteworthy that those with the lowest and highest robustness (*wwm1*, *tir3*, and *met28*, respectively) did align, especially when restricting the analysis to CPSr.

### The beer perturbation space (BPS) has an overall negative impact on robustness

**Fig. 4:**
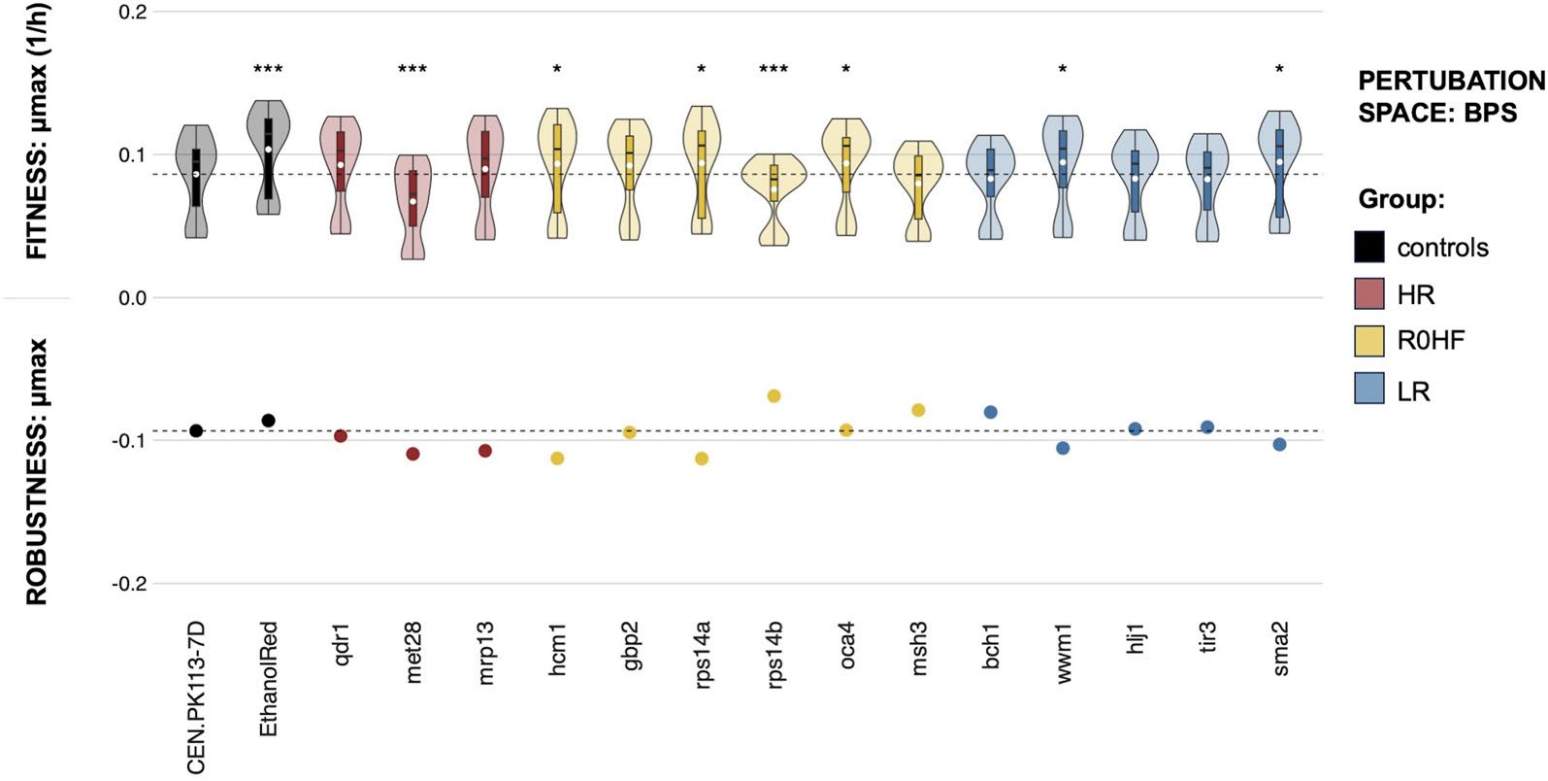
Fitness and robustness in the BPS (beer perturbation space). The y-axis (0;0.2) represents the maximum specific growth rate (1/h) across all strains (controls and mutants with single gene deletions) and perturbations (n = 48), with the black line in the boxplot indicating the median of the distribution and the white dot indicating the mean. Significant difference between the parental strain and the mutants is denoted by p-values obtained from Wilcox tests (*p < 0.05, ***p < 0.001, not significant scores are omitted). Robustness of the maximum growth rate is shown by dots on the y-axis (− 0.2;0), while the horizontal black line marks robustness and fitness of the CEN.PK113-7D parental strain. Groups are colored differently based on analysis of the reference dataset (HR: highest robustness score different from zero; R0HF: robustness equal to zero and highest fitness score; LR: lowest robustness score; controls: CEN.PK113-7D parental and Ethanol Red)

Next, we subjected the yeast mutants to 16 distinct conditions intended to simulate beer fermentation, collectively referred to as BPS. These conditions encompassed various combinations of malts, hops, aromas, and finings. None of the conditions tested inhibited growth entirely (Fig. 4, all data > 0); however, amber malt significantly affected the specific growth rate in all strains, probably due to inhibitory effects by Maillard compounds [21]. Instead, specific aromas, hops or fining agents did not cause a significant change in the maximal specific growth rate with respect to the control strain. In line with its performance in the CPS (Fig. 3), *met28* exhibited a significant drop in fitness also in the BPS (Fig. 4). A similar decrease was observed for *rps14b*, highlighting a trend common to several mutants in terms of reduced mean specific growth rate.

Robustness of the specific growth rate differed between the BPS and CPS. In the former, most gene deletions led to a minor reduction in robustness, with *hcm1* achieving the lowest score. Interestingly, deletion of *rps14b* resulted in the highest robustness, in contrast to its paralog *rps14a*, whose robustness was as low as that of *hcm1* (R = − 0.11). Both *msh3* and *bch1* mutants displayed a slight increase in robustness compared to the parental strain. Robustness values expected from CPSo analysis were not reproduced in this space (Figure S4).

### The lignocellulose hydrolysate perturbation space (LHPS) has an overall positive impact on robustness

**Fig. 5:**
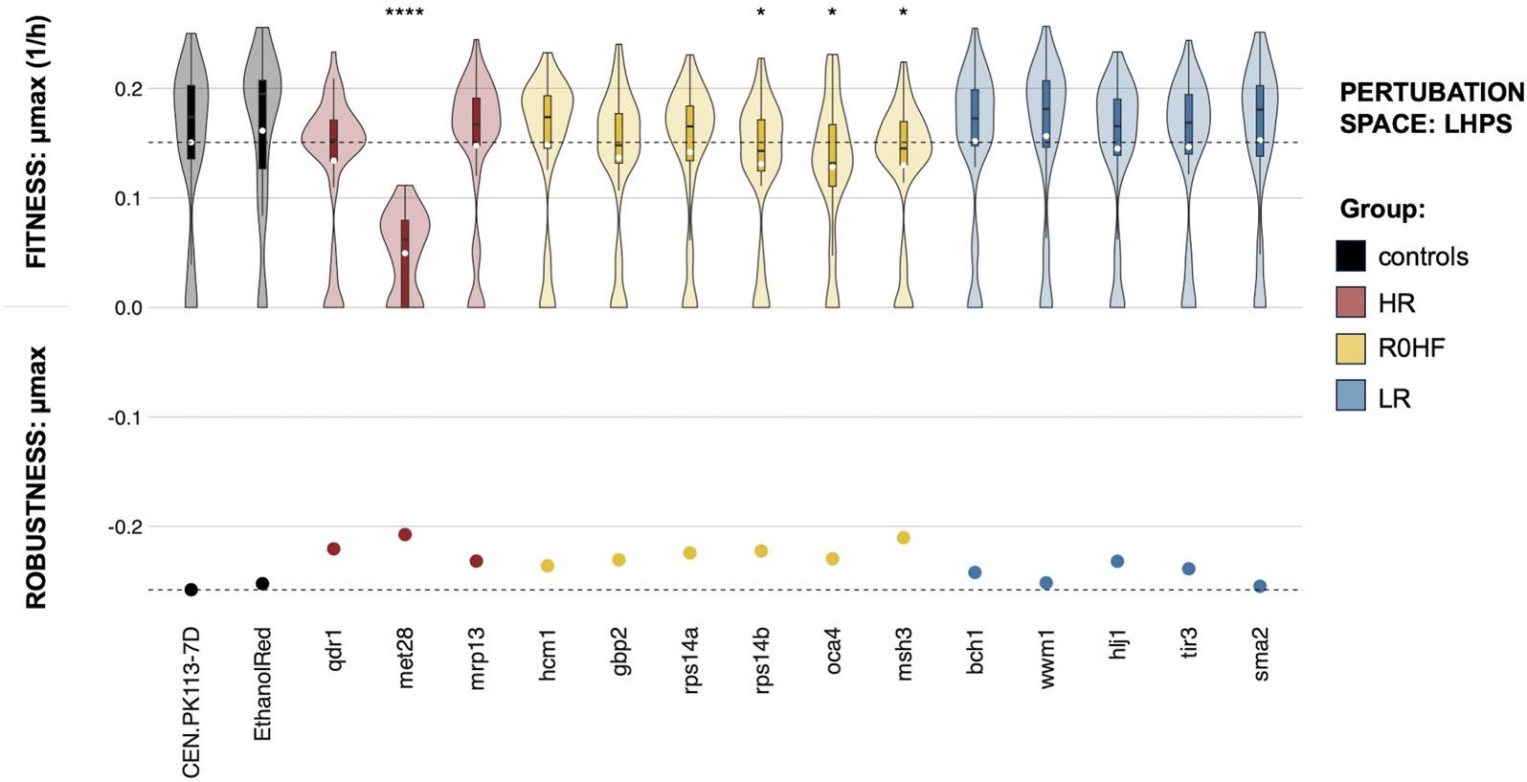
Fitness and robustness in LHPS (lignocellulose hydrolysate perturbation space). The y-axis (0;0.2) represents the maximum specific growth rate (1/h) across all strains (controls and mutants with single gene deletions) and perturbations (n = 48), with the black line in the boxplot indicating the median of the distribution and the white dot indicating the mean. Significant difference between the parental strain and the mutants is denoted by p-values obtained from Wilcox tests (*p < 0.05, ****p < 0.0001, not significant scores are omitted). Robustness of the maximum growth rate is shown by dots on the y-axis (− 0.2;0), while the horizontal black line denotes robustness and fitness of the CEN.PK113-7D parental strain. Groups are colored differently based on analysis of the reference dataset (HR: highest robustness score different from zero; R0HF: robustness equal to zero and highest fitness score; LR: lowest robustness score; controls: CEN.PK113-7D parental and Ethanol Red)

The third perturbation space tested mimicked lignocellulosic hydrolysates and is referred here as LHPS. Its content of acids, aldehydes, alcohols, salts, sugars, and synthetic hydrolysates simulated the fermentation of lignocellulosic hydrolysates into ethanol. Mutants with the highest robustness and fitness (R0HF) showed an overall lower mean fitness compared to the parental strain (Fig. 5). Two conditions stood out as particularly challenging: Delft with 100 g/L ethanol and Delft with pH 3. None of the mutants were able to grow in these extreme environments. Indeed, there were significantly more mutants whose specific growth rate was equal to zero in the LHPS than in the BPS or CPS, likely due to the harsh conditions above.

Ethanol Red exhibited the highest specific growth rate, while *met28* showed the lowest (0.16 and 0.06 1/h respectively). Only four mutants (*msh3, oca4, rps14b*, and *met28*) displayed a notably different fitness distribution in the LHPS when compared to the CEN.PK113-7D parent (Fig. 5). While *msh3*, *oca4*, and *rps14b* presented a strong fitness in the CPSo (Figure S4), their fitness in the LHPS was significantly lower compared to the parental strain. In contrast, the low mean fitness of *met28* was consistent across perturbation spaces.

All tested mutants displayed higher robustness compared to the parental strain (Fig. 5). In particular, *met28* and *msh3* were among the mutants with the highest robustness; whereas *bch1*, *sma2*, *wwm1*, *hlj1*, and *tir3* had relatively low robustness. On the one hand, *met28* exhibited a similar trend in the CPS but an opposite one in the BPS. On the other hand, *sma2* achieved the highest robustness in the CPS, but ranked among the lowest in the LHPS. Generally, the outcome from the reference dataset was confirmed in the LHPS.

### Ethanol red, sma2, and met28 present the highest robustness in all perturbation spaces

After calculating the specific robustness and fitness for each of the tested perturbation spaces, we derived an overall fitness and robustness value, with which to determine the combined response to these different environments.

**Fig. 6:**
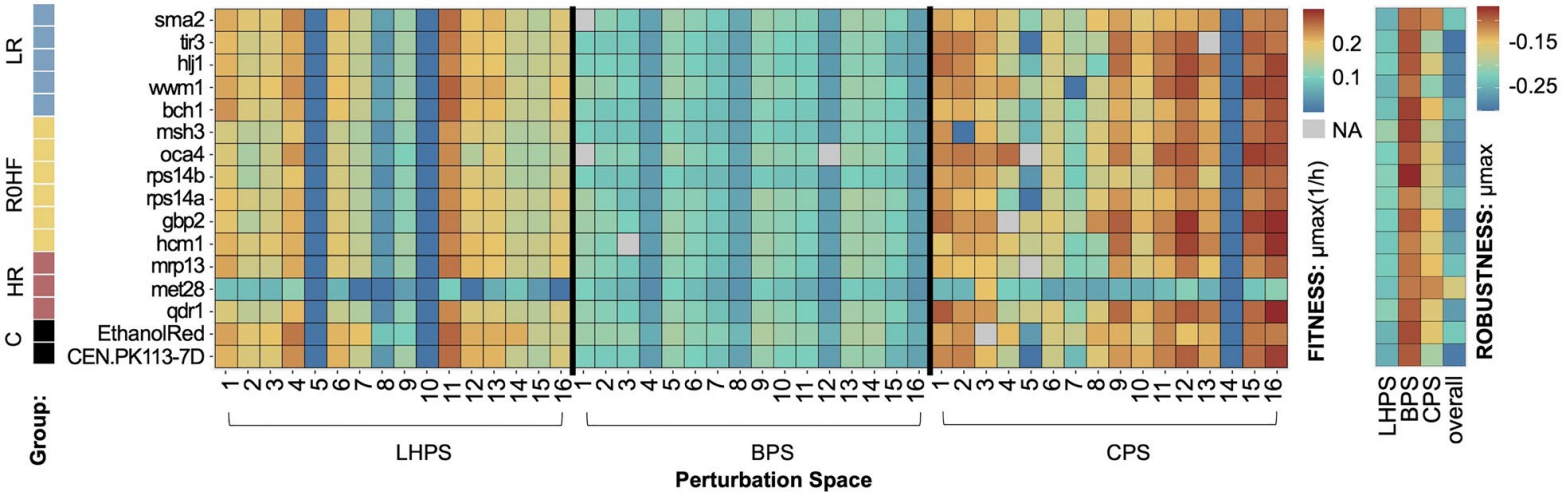
Fitness and robustness of the maximum specific growth rate (1/h) for all tested strains (controls and mutants with single gene deletions) (y-axis) and perturbations (x- axis). Colors of the tiles are assigned based on the minimum and maximum value (legend on the right). The three perturbation spaces are shown on the x-axis divided by a vertical black line: lignocellulose perturbation space (LHPS), beer perturbation space (BPS), and the reference perturbation space (CPS). The four columns on the right denote the robustness of each strain in each perturbation space (x-axis). The color code on the left is representative of the strain listed on the y-axis (HR: highest robustness score different from zero; R0HF: robustness equal to zero and highest fitness score; LR: lowest robustness score; C: CEN.PK113-7D parental and Ethanol Red)

Several conditions resulted in the lowest specific growth rate, including Delft pH 3 (LHPS_5), Delft + 100 g/L ethanol (LHPS_10), Delft + 80 g/L NaCl (LHPS_8; except for Ethanol Red, which had a slightly higher value), and Delft + 50 μg/mL Nigericin sodium salt (CPS_14) (Fig. 6). The *met28* mutant displayed the lowest maximum specific growth rate under all conditions except CPS_3 (YPD), whereby its growth was comparable to that of other strains. Hence, the reduced fitness of *met28* may be related to the composition of Delft minimal medium rather than the specific perturbations applied. CPS_2 (Delft + 20 g/L sucrose) prevented growth of *msh3*, and CPS_7 (Delft + 30 μg/mL Benomyl) that of *wwm1*. CPS_5 (Delft + 1 M sorbitol) impacted primarily the control strains, *tir3*, *rps14a*, and *rps14b*.

Overall, the BPS led to a lower maximum specific growth rate compared to the other two perturbation spaces. This difference can be attributed to the high concentration of less accessible sugars in malt. On the contrary, the BPS resulted in some of the highest robustness values; whereas the combination of all three spaces exhibited the opposite trend (Fig. 6). The *met28* mutant showed the highest robustness in all perturbation spaces, while *wwm1* and *tir3* the lowest.

**Fig. 7:**
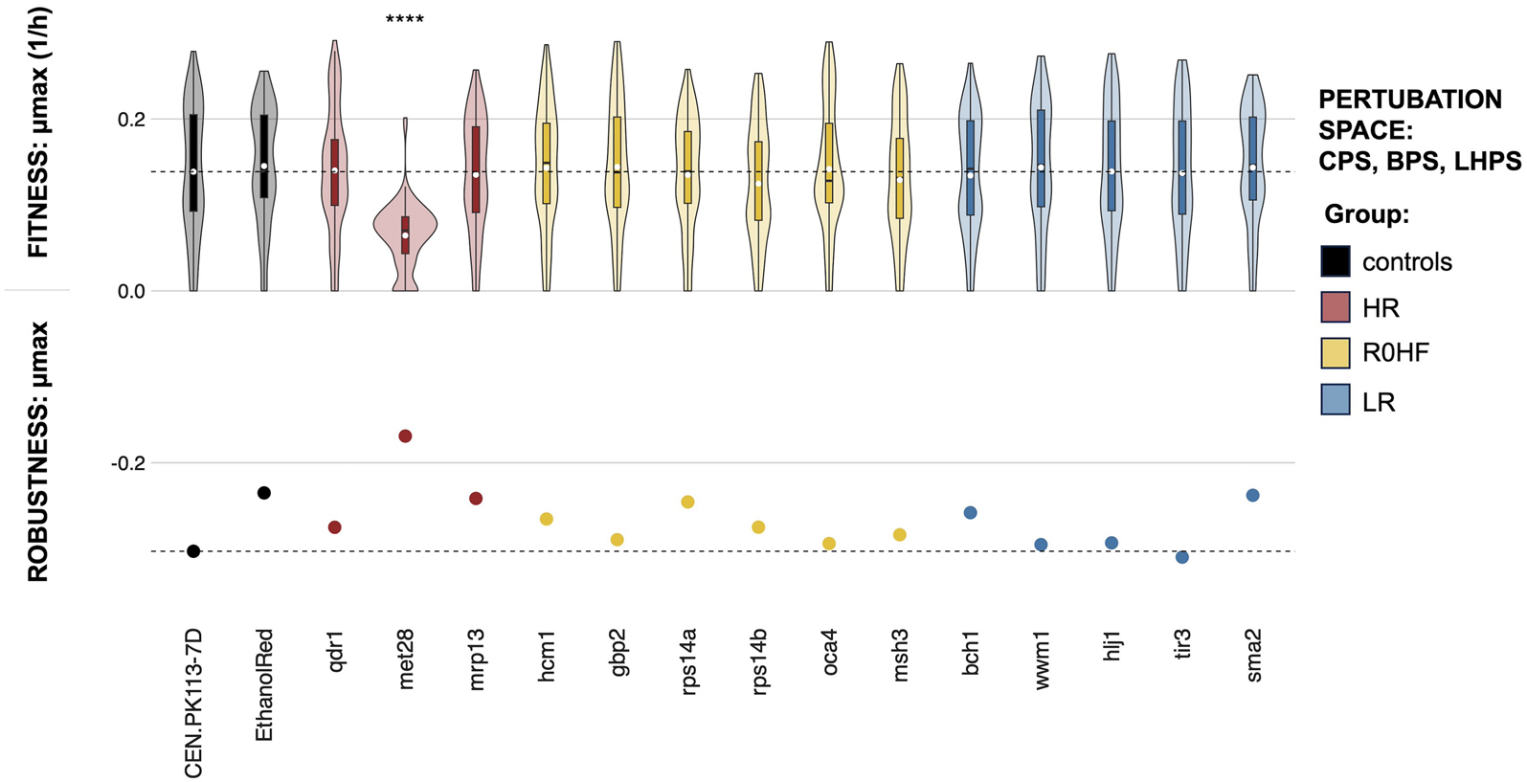
Fitness and robustness in the three perturbation spaces combined together (CPS, BPS, and LHPS). The y-axis (0;0.2) represents the maximum specific growth rate (1/h) across all strains (controls and mutants with single gene deletions) and perturbations (n = 144), with the black line in the boxplot indicating the median of the distribution and the white dot indicating the mean. Significant difference between the parental strain and the mutants is denoted by p-values obtained from Wilcox tests (****p < 0.0001, not significant scores are omitted). Robustness of the maximum growth rate is shown by dots on the y-axis (− 0.2;0), while the horizontal black line denotes robustness and fitness of the CEN.PK113-7D parental strain. Groups are colored differently based on analysis of the reference dataset (HR: highest robustness score different from zero; R0HF: robustness equal to zero and highest fitness score; LR: lowest robustness score; controls: CEN.PK113-7D parental and Ethanol Red)

The fitness distribution of the tested strains resembled that of the parental strain. Only *met28* presented a significantly different distribution and mean, in line with results from single perturbation spaces (Fig. 7). Ethanol Red exhibited the highest fitness, but it did not vary significantly compared to CEN.PK113-7D, despite its prior benchmarking for ethanol production from various substrates, including molasses and corn [32]. The elevated robustness of *met28* (Fig. 7) could be attributed to its overall lower fitness. A correlation between lower fitness and higher robustness has been consistently observed in previous studies [88, 91]. Whereas *sma2* displayed one of the highest values of robustness, followed by *mrp13* and *rps14a*; the robustness of *tir3* was lower than in the parental strain. Except for *met28* or *tir3*, the robustness outcome expected from the reference dataset was not fully replicated across the three perturbation spaces or upon their combination.

## Discussion

In the present study, a previously published dataset containing fitness data of thousands of yeast mutants was analyzed to extract information on genes and metabolic processes associated with robustness. Non-essential gene deletions connected with high or low robustness scores in the reference dataset were replicated in the laboratory CEN.PK113-7D strain. The mutants bearing single non-essential gene deletions were tested in three perturbation spaces simulating relevant industrial processes and robustness of the maximum specific growth rate was investigated. Only some of the non-essential gene deletions (e.g., *met28* or *tir3*) exhibited the same outcome in the tested perturbation spaces as in the reference dataset. The differences observed in comparison to the reference dataset encompasses multiple factors, among which the different strain background (CEN.PK 113-7D versus BY4741) and the different cultivation methodology (solid versus liquid). Furthermore, the perturbation space plays a fundamental role when quantifying the robustness of a system, as different robustness patterns may arise for each space. Nevertheless, we were able to identify four non-essential genes with a consistent impact on robustness across perturbation spaces.

Here, we show for the first time how a dataset containing phenotypic data can be used as input for microbial robustness quantification across different environments. Specifically, the dataset used in this study had two important features that were attractive for robustness quantification [22]. First, it contained fitness data from many different conditions, which is an important requirement when evaluating the significance of the robustness score. Second, the candidates employed in the study featured either essential gene mutations or non-essential gene deletions, thus allowing us to relate the computed robustness to a wide range of individual genes. By calculating robustness from a genetic screen, it is possible to single out robust strains, whose fitness may not be generally as high. Currently, the preferred strategy is to engineer a strain for increased fitness rather than to enhance robustness, both due to lacking methods for enhancing robustness and to a higher priory put to enhancing microbial performance.

Yeast knockout collections have found extensive use in diverse applications, including investigating stress response mechanisms, drug effects, and functional genomics [41, 43, 44, 66, 93]. Here, we identified growth defects associated with specific conditions. For example, Delft medium mixed with 20 g/L sucrose (CPS_2) severely hindered growth of the *msh3* mutant. MSH3 is involved primarily in repairing large insertion/deletion mismatches and no records point to its involvement in sugar or sucrose metabolism. However, a study by VanderSluis et al. [93] indicated an inhibitory effect of galactose on growth of *msh3* mutants, suggesting that a similar mechanism might be at play here. In the case of sorbitol supplementation (CPS_5), twelve out of fourteen gene deletions (e.g., *gbp2* and *sma2*) resulted in a higher specific growth rate than the parental strain. However, no known interactions were found between any of those genes and the enzymes involved in sorbitol catabolism. These examples point to possible links between metabolic processes and the response to environmental conditions.

Analysis of SAFE network regions associated with fitness and robustness failed to identify any processes strictly related to either of these two properties. However, some non-essential gene deletions stood out for their ability to increase robustness: *met28*, *sma2*, *rps14a*, *rps14b, bch1* and *mrp13*. Among these, *met28* caused the highest increase in robustness, but it also resulted in a substantial drop in fitness. In contrast, the *bch1* mutant did not show any significant changes in fitness, but it displayed an increased robustness. Met28 is part of a transcription factor complex (CBF1-MET4-MET28) that regulates sulfur metabolism [58, 77]. Sulfur metabolism underscores many cellular processes: the sulfur metabolic flux correlates positively with specific growth rate [18, 92], it is essential for the synthesis of the stress response factor glutathione [7, 34, 69, 84], and in DNA replication [14]. As Met28 is correlated with specific growth rate, its gene deletion might consistently inhibit the growth of the strain across different cultivation media. Such consistent inhibition could result in a reduced but similar maximum specific growth rate for many perturbations, leading to an elevated robustness value across perturbation spaces. Therefore, the observed elevated robustness might be due to growth inhibition rather than inherent robustness mechanisms. BCH1 belongs to the ChAPs family, which participates in the transport of specific cargo proteins from the Golgi to the plasma membrane [6, 72, 89].

A decrease in robustness was detected upon deletion of TIR3 and WWM1. TIR3 encodes a mannoprotein, which is required during anaerobic growth [2] and sterol uptake [56]. Mannoproteins enable communication with the extracellular environment and contribute to the inertness of the cell surface by providing an external mannan layer [62]. Tir3 belongs to the SRP1/TIP1 protein family, which can be induced under various stress conditions, such as temperature or glucose shock [55, 94]. The present study indicates that mannoproteins might also play a role in robustness. The function of Wwm1 remains unclear, but evidence points to its involvement in apoptosis and metacaspase function [49, 83]. Deletion of WWM1 has shown to impact key aspects of lifespan regulation, which could explain the observed reduced robustness. Crucially, Wwm1 interacts with metacaspases, which contain sulfur ammino acids.

The present work aimed to find specific genetic markers for microbial robustness by investigating the effect of gene deletions on both fitness and robustness in different perturbation spaces. Even if the non-essential gene deletions identified in the reference dataset did not influence robustness in the same way in the three tested perturbation spaces, we were able to confirm and suggest some of the deletions as candidates for further studies on robustness. Mechanistically, our results suggest that sulfur metabolism and sulfur-containing ammino-acids might be crucial for ensuring robustness in yeast. Deletion or overexpression of a single gene might not be sufficient to increase robustness; however, future studies on MET28, BCH1, WWM1, and TIR3, for example, could reveal a synergistic function of these genes impacting robustness. Moreover, understanding sulfur metabolism and mannoproteins will amplify our knowledge of the metabolic processes affecting overall cell robustness. Expansion of the perturbation space, systems, and most crucially measured parameters is necessary to explore processes that could not be highlighted in this study. Finally, we here demonstrate that phenomics datasets are a key tool to investigate complex and understudied mechanisms.

## Conclusions

In this study, we show how robustness analysis can be applied to phenomics datasets and coupled with metabolic information to obtain an overall map of robustness in terms of both, environmental and genetic landscapes. This example can guide the integration of robustness in the engineering of cell factories, thus complementing the current focus on fitness and production.

## Material and methods

### Robustness quantification using a previously published dataset

A publicly accessible dataset by Costanzo et al. [22] provided the starting point for the present study. In particular, we focused on two key sets of data. The initial set contained single-mutant fitness data in the form of normalized colony size, across 14 different conditions. This set covered all non-essential deletion and essential temperature-sensitive mutant arrays, totaling 4429 unique mutants derived from BY4741 or Y7092 strains [11]. The second set provided insights into genes and the related Spatial Analysis of Functional Enrichment (SAFE) annotations. First, these data were imported and processed in R. Throughout our analysis, we employed specific R libraries to facilitate various aspects of data manipulation, visualization, and exploration [4, 12, 24–31, 74, 76, 80, 87, 98]. Fitness distributions across strains for each condition were compared in R using analysis of variance (Alboukadel [4]). Robustness of the normalized colony size was calculated across 14 environments using Eq. 1 [90] where the index of dispersion ($\frac{{\sigma }^{2}}{\overline{x} }$) calculated with a set of phenotypic data is normalized by the mean “*m*” of the data across all strains:

1 $$R=-\frac{{\sigma }^{2}}{\overline{x}}\frac{1}{m }$$

The set of 14 conditions corresponded to the perturbation space, the yeast strains to the system, and the normalized colony size to the cellular function (phenotype). Yeast strains were ranked based on two criteria: fitness and robustness in response to different perturbations. To generate datasets with mutants with either low and high robustness and fitness, the mutants belonging to 10th and 90th percentiles were extracted from the original dataset. Data from the SAFE network regions were merged with the first dataset based on gene and allele names. Once the metabolic processes were associated with each gene and allele, 14 mutants with non-essential gene deletions were picked among the ten with either high robustness, low robustness or robustness equal to zero and highest fitness (Table 1), and categorized into three groups: (I) three mutants with notably high robustness distinct from zero: HR; (II) six mutants with both the highest fitness and a robustness value of zero: R0HF; and (III) five mutants with the lowest robustness: LR. The 14 genes were picked belonging to different SAFE regions. A script with in line description is available on Github (https://github.com/cectri/rob_genetic_markers).

### Strains and gene deletions using CRISPR-Cas9

To test whether robustness calculated from the Costanzo dataset could be reproduced in other perturbation spaces, and if specific gene deletions affected robustness, the selected non-essential genes (Table 1) were deleted from the laboratory CEN.PK113-7D strain (MATa URA3 HIS3 LEU2 TRP1 MAL2-8c SUC2) [37]. Gene deletions were carried out using the LiAc/salmon sperm carrier DNA/polyethylene glycol method [45] in combination with CRISPR/Cas9 for improved integration efficiency [3]. YN2_1_Cas9 was the backbone Cas9 plasmid used in this study (bearing both the Cas9 and single guide RNA—sgRNA expression cassettes) and was previously developed in our laboratory [16]. In the sgRNA expression cassette, a GFP-dropout insert was designed to be replaced with the gene-specific 20-bp sgRNA.

To design the sgRNAs required for gene deletions and the donor DNAs needed for transformations, a script in R was developed for automated and fast design. The script is available on GitHub (https://github.com/lucatorep/sgRNA_design_scripts).

To determine the sgRNA sequences, the R script compared and found the best sequences in CRISPR-ERA [61], Yeast CRISPRi [79], and CHOPCHOP [59]. Briefly, sgRNAs were selected using the following parameters: (I) an ATAC-seq value above 0.7; (II) a nucleosome presence value below 0.2; (III) absence of poly-nucleotide sequences (more than 4 identical nucleotides in a row) and off-targets; (IV) CG content above 25%; (V) sgRNA sequence present in multiple databases, and (VI) prediction of sgRNA efficiency above 50%.

To completely remove the coding sequence of the gene selected for deletion, an 11-bp sequence comprising three stop codons in different frames (TAACTAGCTGA) was flanked by 50-bp homology arms in the promoter and terminator regions of the selected gene. Homology arms were automatically selected from the R script mentioned above. Therefore, the final donor DNA was 111 bp long (Table S2).

All oligos for each gene (sgRNAs and donor DNAs) were ordered as single-stranded (forward and reverse) oligonucleotides from Eurofins. The sgRNAs already contained sticky ends suitable for assembly in the YN2_1_Cas9 vector (Table S3). The generation of double-stranded oligonucleotides (both sgRNAs and donor DNAs), and insertion of sgRNA into YN2_1_Cas9 were carried out as described previously (REF). The transformation (Gietz, 2014) included an 18 min heat shock, as well as 1 μg of double-stranded donor DNA plus 300 ng of Cas9-sgRNA plasmid for each gene. Transformants were then plated on YPD + G418 plates and incubated for 3 days at 30 °C. Colonies were verified by colony PCR using gene-specific oligos (Table S4). The Cas9 plasmid was cured by re-streaking positive clones twice on antibiotic-free YPD plates.

### Composition of the three perturbation spaces

Three perturbation spaces were tested in the study. The first perturbation space, CPS, included conditions similar to those used by Costanzo et al. [22] (Additional material). The Delft minimal medium used as base for all 16 conditions contained 5 g/L (NH_4_)_2_SO_4_, 3 g/L KH_2_PO_4_, 1 g/L MgSO_4_∙7H_2_O, 1 mL (in 1 L) of trace metals solution (Table S5), and 1 mL (in 1 L) of vitamin solution (Table S6). The medium was adjusted to pH 5 with NaOH and buffered with 100 mM C_8_H_5_KO_4_. The medium was supplemented with different sugars and antifungal agents (Table 2).

The other two perturbation spaces mimicked industrial processes that employ *S. cerevisiae* as a cell factory to produce valuable chemicals or fermentation products. First, the BPS was composed of 16 conditions, each corresponding to a mixture of malt, hops, an aroma, and a fining agent (Tables 3, 4). The malt was dissolved in water according to the vendor’s instructions (1 kg liquid or solid malt in 8 L water) and mixed with a magnetic stirrer for 10 min. To avoid any precipitation in culture plates, the dissolved malt was centrifuged at 5000 rpm for 5 min and the liquid fraction was filter-sterilized, first through a 2.5 µm cellulose filter and then through a 0.2 µm PES filter. To prepare the hops (2.6 g/L), we chose the boiling time in distilled water based on the suppliers’ recommendations. Hops with a high content of alpha acids (bittering hops), including Simcoe, Citra, and Sabro HBC 438, were boiled for 60 min. Perle, Fuggle, and Nelson Sauvin late-hops were boiled for 15 min. Post-boil Amarillo, Mosaic, and Cascade hops were added after boiling and left for 20 min. Continuous hopping was also used with Magnum, East-Kent, and Tettnanger hops, which were added in 1/3 proportions every 20 min for a total time of 1 h. Hops were cooled down, aromas were added to the hop mixture (620 μL in 200 mL), and the latter was filter-sterilized. Enzymes (0.5 g/L) were also dissolved in water and filter-sterilized. Malts and hops were mixed with aromas and fining agents in a 1:1 ratio.

**Table 3:** List of malts, hops, fining agents, and aromas used in the BPS

*Malts* Spraymalt Extra Light (Muntons), Spraymalt Wheat (Muntons), Coobra liquid malt extract light, TC Malt Extract Amber (Coopers)	
*Hops pellets* Perle (Germany), Fuggle (England), Simcoe Humle (USA), East-Kent Gold (England), Nelson Sauvin Humle (New Zealand), Cascade (USA), Magnum Humle (Germany), Citra (USA), Mosaic (USA), Amarillo (USA), Tettnanger (Germany), Sabro Brand HBC 438 (USA)	
*Aromas* Vanilj Vodka (Strands), Pepparmint (Strands), Mia Theresa Essens (Strands), Svartvinbärsbrännvin (Strands), Citron Vodka (Strands), Grappa Stravecchia (Strands)	
*Finings* Distiller’s Enzyme Alpha-Amylase (StillSpirits), Distiller’s Enzyme Glucoamylase (StillSpirits)

**Table 4:** List of conditions in the BPS

Perturbation code	Malt	Hop	Aroma	Fining agent
BPS_1	Spraymalt extra light	Perle	Vanilj vodka	/
BPS_2	Spraymalt wheat	Fuggle	Vanilj vodka	/
BPS_3	Coobra light	Simcoe	Peppermint	/
BPS_4	Amber malt	East-Kent Gold	Peppermint	/
BPS_5	Spraymalt extra light	Nelson Sauvin	Svartvinbärsbrännvin	/
BPS_6	Spraymalt wheat	Cascade	Svartvinbärsbrännvin	/
BPS_7	Coobra light	Magnum	Mia Theresa Essens	/
BPS_8	Amber malt	Citra	Mia Theresa Essens	/
BPS_9	Spraymalt extra light	Mosaic	Citron Vodka	/
BPS_10	Spraymalt wheat	Amarillo	Citron Vodka	/
BPS_11	Coobra light	Tettnanger	Grappa Stravecchia	/
BPS_12	Amber malt	Sabro brand HBC 438	Grappa Stravecchia	/
BPS_13	Spraymalt extra light	/	/	Glucoamylase
BPS_14	Spraymalt wheat	/	/	Glucoamylase
BPS_15	Coobra light	/	/	Alpha-amylase
BPS_16	Amber malt	/	/	Alpha-amylase

The LHPS included components commonly encountered during lignocellulose hydrolysate fermentation. The 16 test media were prepared by mixing specific inhibitors or sugars with the other Delft ingredients (concentration of Delft components as in CPS) (Table 5). Stock solutions of the specific inhibitors were adjusted to pH 5 and filter-sterilized separately using a 0.2-µm PES filter. Synthetic hydrolysate components were also mixed with Delft medium.

**Table 5:** List of conditions in the LHPS

Perturbation code	Carbon source	Antifungal agent
LHPS_1	Glucose 20 g/L	/
LHPS_2	Glucose 5 g/L	/
LHPS_3	Glucose 100 g/L	/
LHPS_4	Glucose 20 g/L	no buffer
LHPS_5	Glucose 20 g/L	pH = 3
LHPS_6	Glucose 20 g/L	Half concentration of vitamins and trace metals
LHPS_7	Glucose 20 g/L	NaCl 25 g/L
LHPS_8	Glucose 20 g/L	NaCl 80 g/L
LHPS_9	Glucose 20 g/L	Ethanol 50 g/L
LHPS_10	Glucose 20 g/L	Ethanol 100 g/L
LHPS_11	Glucose 20 g/L	Acetic acid 2 g/L
LHPS_12	Glucose 20 g/L	Formic acid 2 g/L
LHPS_13	Glucose 20 g/L	Lactic acid 10 g/L
LHPS_14	Glucose 20 g/L	5-(hydroxymethyl)furfural 1 g/L
LHPS_15	/	Spruce synthetic hydrolysate^a^ 20%
LHPS_16	/	Corn synthetic hydrolysate^a^ 100%

^a^Composition of the synthetic hydrolysates can be found in Table S7

### Strain cultivation

Briefly, 10 μL of the strain’s glycerol stock was inoculated in 5 mL Delft 2% glucose. The pre-culture was incubated overnight at 30 °C and 200 rpm shaking. Then, the strains were reinoculated in exponential phase in 250 μL medium at a starting OD600 of 0.02. Strains were grown in triplicates in 96-well plates (CR1496dg, Enzyscreen). Given the 16 different conditions in the perturbation space, two strains were accommodated on each plate. For LHPS and BPS plates, a cover (CR1296, Enzyscreen) was applied to minimize oxygen diffusion, effectively creating anaerobic conditions. Conversely, for CPS plates, a two-step approach was employed. First, a clear polyester adhesive film was applied to prevent contamination of the covers with any potentially toxic compounds caused by splashing. Second, an aerobic cover (CR1396b) was placed on top of the film. The cultivation process was monitored by measuring green values using a Growth Profiler 960 (Enzyscreen). All experiments were conducted at 30 °C with continuous shaking at 250 rpm for a duration of 48 h. The green values obtained were subsequently utilized for in-depth analysis of fitness and robustness.

### Fitness and robustness assessment

To evaluate fitness, the maximum specific growth rate (1/h) was estimated for each well using the ‘‘ all_splines’’ function in R. In the case of no growth, the maximum specific growth rate was set to zero. In cases where the ‘‘all_splines’’ function failed to adequately fit the growth curve (with an R-squared value < 0.99), the maximum specific growth rate was designated as NA. The calculation of robustness was carried out using Eq. 1. When calculating robustness for each perturbation space, all replicates were considered collectively. Consequently, no mean robustness or standard error was computed. The entire data analysis process and the plots were generated using R Studio Version 2023.06.2 + 561. The scripts along with in-line descriptions and the raw data are readily accessible via GitHub at the following link https://github.com/cectri/rob_genetic_markers.

### Supplementary Information

Additional file1. Table S1. SAFE network regions associated with mutants characterized by either high or low fitness or robustness. Table S2. Donor sequences used in this study. Table S3. Details of sgRNAs used in this study. Table S4. Oligos for sgRNAs used in this study. Table S5. Composition of the trace metals solution. Table S6. Composition of the vitamin solution. Table S7. Composition of spruce and corn synthetic hydrolysates. Figure S1. Distribution of fitness and robustness of the 10th and 90th percentiles of the original dataset. Figure S2. SAFE regions mean fitness and robustness (reference dataset). Figure S3. Fitness and robustness in the CPSr. Figure S4. Fitness and robustness in the CPSo.

## Data Availability

The datasets and scripts supporting the conclusions of this article are published in the following repositories: https://github.com/cectri/rob_genetic_markers. https://github.com/lucatorep/sgRNA_design_scripts
