# Supplementary material for: Robustness quantification of a mutant library screen revealed key genetic markers in yeast
Source: Microb Cell Fact. 2024 Aug 4;23:218. doi: 10.1186/s12934-024-02490-2 (PMC11298085; doi:10.1186/s12934-024-02490-2)
Supplement: Supplementary file 1 — Additional file1. Table S1. SAFE network regions associated with mutants characterized by either high or low fitness or robustness. Table S2. Donor sequences used in this study. Table S3. Details of sgRNAs used in this study. Table S4. Oligos for sgRNAs used in this study. Table S5. Composition of the trace metals solution. Table S6. Composition of the vitamin solution. Table S7. Composition of spruce and corn synthetic hydrolysates. Figure S1. Distribution of fitness and robustness of the 10th and 90th percentiles of the original dataset. Figure S2. SAFE regions mean fitness and robustness (reference dataset). Figure S3. Fitness and robustness in the CPSr. Figure S4. Fitness and robustness in the CPSo. [file 12934_2024_2490_MOESM1_ESM.pdf]

Robustness quantification of a mutant library screen revealed key genetic markers in yeast

Cecilia Trivellin[1], Luca Torello Pianale[1], Lisbeth Olsson[1*]

[1]Department of Life Sciences, Division of Industrial Biotechnology, Chalmers University of Technology, Gothenburg, 412 96, Sweden

*corresponding author

Additional material

Table of contents:

Tables S1–S7

Figures S1–S4

Table S1. SAFE network regions associated with mutants characterized by either high or low fitness or robustness (the 10th or 90th percentiles).

**SAFE network region**	**High Robustness**	**Low Robustness**	**High Fitness**	**Low Fitness**
cell polarity	62	35	67	35
NA	35	23	16	23
glycosylation, cell wall biosynthesis	32	43	26	43
transcription	27	36	11	36
mitosis	22	38	15	38
vesicle traffic	17	26	3	26
mitochondria	14	27	16	27
DNA replication/repair	9	23	7	23
mRNA processing	9	20	8	20
metabolism	6	8	2	8
nuclear transport	6	14	7	14
rRNA processing	6	34	3	34
protein turnover	4	18	7	18
peroxisome	3	2	2	2
ribosome biogenesis	3	20	3	20
tRNA wobble modification	3	7	2	7
MVB sorting	1	7	3	7
cytokenesis	1	NA	NA	NA

*n varies among groups as not all mutant strains were associated with a SAFE region

Table S2. Donor sequences used in this study. These oligos were annealed and then used as donor DNA to delete the targeted gene (found in the oligo code). Lowercase letters refer to the homology arms for homologous recombination, while uppercase letters refer to the barcode used to replace the coding sequence.

Oligo Code	Sequence (5' – 3')
LT202_GBP2_Donor_FW	aggtgaaaaaaattgaaaaaaaatgaaagcttggatgaaatcaattatatTAACTAGCTGAttatggtggttgtagtttacagatctcttatgctagacgtgattaattcc
LT202_GBP2_Donor_RV	ggaattaatcacgtctagcataagagatctgtaaactacaaccaccataaTCAGCTAGTTAatataattgatttcatccaagctttcatttttttcaatttttttcacct
LT203_RPS14A_Donor_FW	gcttcctcaggtagacagttgaaatgaattggttattaatcactatatatTAACTAGCTGAagactccaccagaaagaagggtggtagaagaggtagaagattatgagtta
LT203_RPS14A_Donor_RV	taactcataatcttctacctcttctaccacccttctttctggtggagtctTCAGCTAGTTAatatatagtgattaataaccaattcatttcaactgtctacctgaggaagc
LT204_HCM1_Donor_FW	attctattcttttcccccttttattatagttacatctactatttgagctTAACTAGCTGAttatcataatcacccttccaacgatagcggtaatgaaaagaattgacttt
LT204_HCM1_Donor_RV	aaagtcaattcttttcattaccgctatcgttggaagggtgattatgataaTCAGCTAGTTAagctcaaatagtagatgtaactataataaaagggggaaaagaatagaat
LT205_MSH3_Donor_FW	acgtgaattttcaatgataaataagctggaacaatggtgataggtaatgaTAACTAGCTGAagcaacggataaactcgcgaaattactatcattggatatccactgatgcg
LT205_MSH3_Donor_RV	cgcatcagtggatatccaatgatagtaatttcgcgagtttatccgttgctTCAGCTAGTTAtcattacctatcaccattgttccagcttatttatcattgaaaattcacgt
LT206_OCA4_Donor_FW	agttttctagcattaaatggacagtaaggtaaagaaatgagatgtggttgTAACTAGCTGAcgttccggaagagcatcacttttatagggaacatatttttacatagttta
LT206_OCA4_Donor_RV	taaactatgtaaaaatatgttccctataaaagtgatgctcttccggaacgTCAGCTAGTTAcaaccacatctcatttctttaccttactgtccatttaatgctagaaaact
LT207_WWM1_Donor_FW	cgagccggcagctggaagaaaaagtttactttagtcatgataatcaaaagTAACTAGCTGAgttcgacggcggatttgatgatggatttgacggtggtgacttttaatgtt
LT207_WWM1_Donor_RV	aacattaaaagtcaccaccgtcaaatccatcatcaaatccgccgtcgaacTCAGCTAGTTActtttgattatcatgactaaagtaaactttttcttccagctgccggctcg
LT208_MRP13_Donor_FW	gaacactttgtcaatgtatttagttgtattcatacccaatttattggcacTAACTAGCTGAggaaacaccacctcctcaggaatccaccgaaaaagaggaaaaatagtacc
LT208_MRP13_Donor_RV	ggtactatttttcctctttttcggtggattcctgaggaggtggtgtttccTCAGCTAGTTAgtgccaataaattgggtatgaatacaactaaatacattgacaaagtgttc
LT209_TIR3_Donor_FW	tggagagtataagcgtttcgtctcctcttcctttctctactgcagattttTAACTAGCTGAtggtgttggtgccgctattgccggtgccgctgctatgctcttatgattgt
LT209_TIR3_Donor_RV	acaatcataagagcatagcagcggcaccggcaatagcggcaccaacaccaTCAGCTAGTTAaaaatctgcagtagagaaaggaagaggagacgaaacgcttatactctcca
LT210_QDR1_Donor_FW	gcaatcagcgttcaaaggaacttattcttgcttaaaaaaagtgataatctTAACTAGCTGAggctggaaggtcagttggtaaaaattcagaaaaagtttcgacgtgaagaa
LT210_QDR1_Donor_RV	ttcttcacgtcgaaactttttctgaatttttaccaactgaccttccagccTCAGCTAGTTAagattatcacttttttaagcaagaataagttcctttgaacgctgattgc
LT211_MET28_Donor_FW	gttgtaggtcactggcgtgagtcaaggccgggcagccaatgactaagaacTAACTAGCTGAtttgaaattgttgaatgacattaagagacggaacatgggcaggtagcccg
LT211_MET28_Donor_RV	cgggctacctgcccatgttccgtctcttaatgtcattcaacaatttcaaaTCAGCTAGTTAgttcttagtcattggctgcccggccttgactcacgccagtgacctacaac
LT212_RPS14B_Donor_FW	aagatatagtcgcaagaacttgccttctacgagtataaaagcggtactatTAACTAGCTGAagactccaccagaaagaagggtggtagaagaggtagaagattatgatttc
LT212_RPS14B_Donor_RV	gaaatcataatcttctacctcttctaccacccttctttctggtggagtctTCAGCTAGTTAatagtaccgcttttatactcgtagaaggcaagttcttgcgactatatctt
LT213_SMA2_Donor_FW	ataccatgcacttacttaaattctatgaacacatccatcaattaaaagatTAACTAGCTGAagaagacacatctaactccccttctccagacttgatggagaagtaattca
LT213_SMA2_Donor_RV	tgaattacttctccatcaagtctggagaaggggagttagatgtgtcttctTCAGCTAGTTAatcttttaattgatggatgtgttcatagaatttaagtaagtgcatggtat
LT214_HLJ1_Donor_FW	gtgaagaacataaaggaagctggcagaagttcttttcatacacctctactTAACTAGCTGAcatctttattgttcttcctatgattaaagattacctgtttagttaaacac
LT214_HLJ1_Donor_RV	gtgtttaactaaacaggtaatctttaatcataggaagaacaataaagatgTCAGCTAGTTAagtagaggtgtatgaaaagaacttctgccagcttcctttatgttcttcac
LT215_BCH1_Donor_FW	cgataatgtagtaacaaatggacccttaaacatctcaattcatcttcttgTAACTAGCTGAtctaaattttctgaagaatttcacgaatgacactttcgataattagaaaa
LT215_BCH1_Donor_RV	ttttctaattatcgaaagtgtcattcgtgaaattcttcagaaaatttagaTCAGCTAGTTAcaagaagatgaattgagatgtttaagggtccatttgttactacattatcg

Table S3. Details of sgRNAs used in this study. No common sgRNAs were found for RPS14A; therefore, the one from CRISPR-ERA was selected.

Gene	Transcript ID	Sequence	Genomic location	GC %	Self compl.[a]	Efficiency[a] (%)	Offtargets[a,b]	PolyN	TSS dist[c]	Nucleosome[c]	Chromatin[c]
MRP13	YGR084C	ATAACAGTGGTGATTAATGA	chrVII:648111	30	0	61.75	0	No	168	0.02	0.87
QDR1	YIL120W	ATAGTAGATAGACCCTGCCA	chrIX:134664	45	0	71.31	0	No	456	0.09	0.71
MET28	YIR017C	GCGGAGAAGAAAGAACACAG	chrIX:383768	50	0	74.52	0	No	348	0	0.79
MSH3	YCR092C	TCCCTTGGACAAACAGGTGA	chrIII:279400	50	0	58.77	0	No	443	0.1	0.89
HCM1	YCR065W	TGGTAAGGGTCATTTCTGGG	chrIII:229834	50	0	75.26	0	No	716	0.13	0.98
WWM1	YFL010C	TGTTGAGGCTGATAGTAACG	chrVI:115454	45	0	73.32	0	No	309	0.19	1
BCH1	YMR237W	AACATCTTCCAACTCACCCG	chrXIII:744437	50	0	72.19	0	No	772	0.09	0.43
OCA4	YCR095C	AAGTATACCAATGACCAACG	chrIII:288758	40	0	80.75	0	No	529	0.04	0.11
TIR3	YIL011W	AGCAGAAACCAATTGAGTGG	chrIX:333770	45	0	65.64	0	No	88	0.63	0.12
GBP2	YCL011C	GGAGGAAGCAGATCATTCAG	chrIII:103087	50	0	69.02	0	No	302	0.09	0.62
RPS14B	YJL191W	TACAAAGGTATCGTTAAAGG	chrX:74259	35	0	65.62	0	No	509	0.4	0.57
HLJ1	YMR161W	TATAAACTCTGAAACCACCG	chrXIII:578206	40	0	75.05	0	No	550	0.1	0.4
SMA2	YML066C	TCGTACCTGACAATTGACAT	chrXIII:141307	40	0	70.23	0	No	240	0.52	0.33
RPS14A	YCR031C	ATCGCTATGAATTACTTTGG	chrIII:177500	35	--	--	0	No	170	0.13	0.12

[a] *from CHOPCHOP, https://chopchop.cbu.uib.no/;*
[b] *from CRISPRERA, http://crispr-era.stanford.edu/ ;*
[c] *from YeastCRISPRi, https://lp2.github.io/yeast-crispri/.*

Table S4. Oligos for sgRNAs used in this study. These oligos were annealed and then cloned into the YN2_1_Cas9 Plasmid. The target sequence for Cas9 is included in the FW oligos. Lowercase letters denote the sticky ends necessary for the restriction-cloning step with the Cas9 plasmid, while uppercase letters refer to the sgRNA sequence. The target gene is specified in the oligo code.

Oligo Code	Sequence (5' – 3')
LT186_MRP13_sgRNA_FW	gactATAACAGTGGTGATTAATGA
LT186_MRP13_sgRNA_RV	aaacTCATTAATCACCACTGTTAT
LT187_QDR1_sgRNA_FW	gactATAGTAGATAGACCCTGCCA
LT187_QDR1_sgRNA_RV	aaacTGGCAGGGTCTATCTACTAT
LT188_MET28_sgRNA_FW	gactGCGGAGAAGAAAGAACACAG
LT188_MET28_sgRNA_RV	aaacCTGTGTTCTTTCTTCTCCGC
LT190_HLJ1_sgRNA_FW	gactTATAAACTCTGAAACCACCG
LT190_HLJ1_sgRNA_RV	aaacCGGTGGTTTCAGAGTTTATA
LT191_SMA2_sgRNA_FW	gactTCGTACCTGACAATTGACAT
LT191_SMA2_sgRNA_RV	aaacATGTCAATTGTCAGGTACGA
LT192_MSH3_sgRNA_FW	gactTCCCTTGGACAAACAGGTGA
LT192_MSH3_sgRNA_RV	aaacTCACCTGTTTGTCCAAGGGA
LT193_HCM1_sgRNA_FW	gactTGGTAAGGGTCATTTCTGGG
LT193_HCM1_sgRNA_RV	aaacCCCAGAAATGACCCTTACCA
LT194_WWM1_sgRNA_FW	gactTGTTGAGGCTGATAGTAACG
LT194_WWM1_sgRNA_RV	aaacCGTTACTATCAGCCTCAACA
LT195_BCH1_sgRNA_FW	gactAACATCTTCCAACTCACCCG
LT195_BCH1_sgRNA_RV	aaacCGGGTGAGTTGGAAGATGTT
LT196_OCA4_sgRNA_FW	gactAAGTATACCAATGACCAACG
LT196_OCA4_sgRNA_RV	aaacCGTTGGTCATTGGTATACTT
LT197_TIR3_sgRNA_FW	gactAGCAGAAACCAATTGAGTGG
LT197_TIR3_sgRNA_RV	aaacCCACTCAATTGGTTTCTGCT
LT198_GBP2_sgRNA_FW	gactGGAGGAAGCAGATCATTCAG
LT198_GBP2_sgRNA_RV	aaacCTGAATGATCTGCTTCCTCC
LT200_RPS14B_sgRNA_FW	gactTACAAAGGTATCGTTAAAGG
LT200_RPS14B_sgRNA_RV	aaacCCTTTAACGATACCTTTGTA
LT201_RPS14A_sgRNA_FW	gactATCGCTATGAATTACTTTGG
LT201_RPS14A_sgRNA_RV	aaacCCAAAGTAATTCATAGCGAT

Table S5. Composition of the trace metals solution.

Chemical	Amount (g/L)
EDTA	0.015
$ZnSO_4 \cdot 7H_2O$	0.0045
$MnCl_2 \cdot 4H_2O$	0.0008
$CoCl_2 \cdot 6H_2O$	0.0003
$CuSO_4 \cdot 5H_2O$	0.0003
$Na_2MoO_4 \cdot 2H_2O$	0.0004
$CaCl_2 \cdot 2H_2O$	0.0045
$FeSO_4 \cdot 7H_2O$	0.003
H_3BO_3	0.001
KI	0.0001

Table S6. Composition of the vitamin solution.

Vitamin	Amount (g/L)
d-Biotin	0.00005
Calcium D(+) pantothenate	0.001
Nicotinic acid	0.001
Myo-inositol	0.025
Thiamine HCl	0.001
Pyridoxine HCl	0.001
Para-aminobenzoic acid	0.0002

Table S7. Composition of spruce and corn synthetic hydrolysates.

Chemical (CAS Number)	Spruce (100%) g/L	Corn (100%) g/L
D-(+)-Glucose (14431-43-7)	34	7.4
D-(+)-Xylose (58-86-6)	9	40.2
L-(+)-Arabinose (5328-37-0)	4	7
D-(+)-Galactose (59-23-4)	4.50	3.34
D-(+)-Mannose (3458-28-4)	0	0.8
Acetic acid (64-19-7)	7.50	2
Levulinic acid (123-76-2)	2.40	0.3
Formic acid (64-18-6)	0	0.7
Furfuryl alcohol (98-00-0)	1.10	0
5-(Hydroxymethyl)furfural (67-47-0)	3.40	0.4
Vanillin (121-33-5)	0.11	0.05

Figure S1.

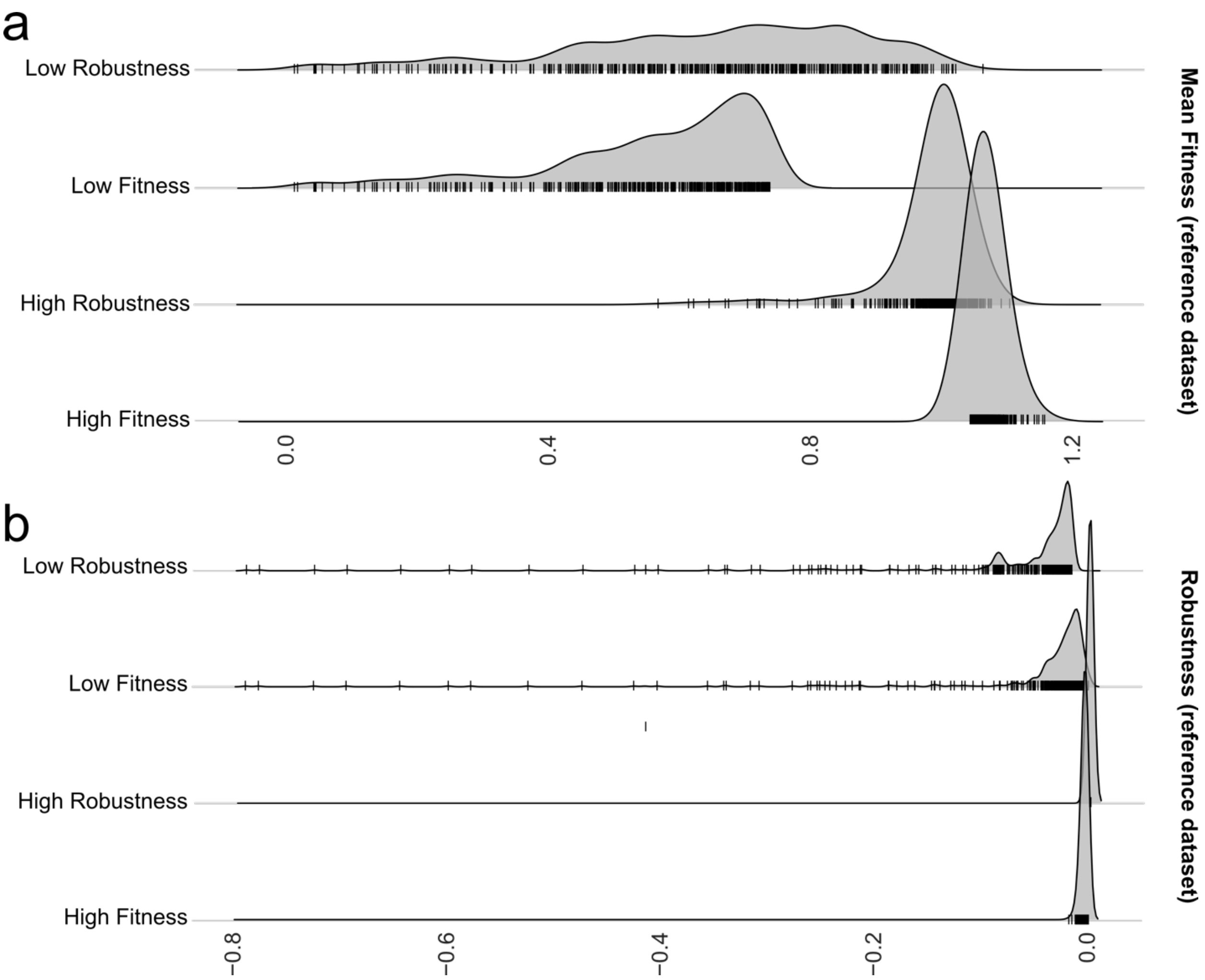

Figure S1. Distribution of fitness and robustness of the 10th and 90th percentiles of the original dataset. *a) The x-axis (0-1.2) represents the mean of the normalized colony size across 14 conditions for all mutants of the reference dataset. Each vertical line corresponds to one mutant's mean fitness. The y-axis represents subsets of data extracted from the reference dataset, using the 10th and 90th percentile cutoffs for both fitness and robustness. b) Robustness of the normalized colony size is shown on the x-axis (-0.8;0). Each vertical black line represents a mutant from the reference dataset with robustness calculated across 14 conditions.*

Figure S2.

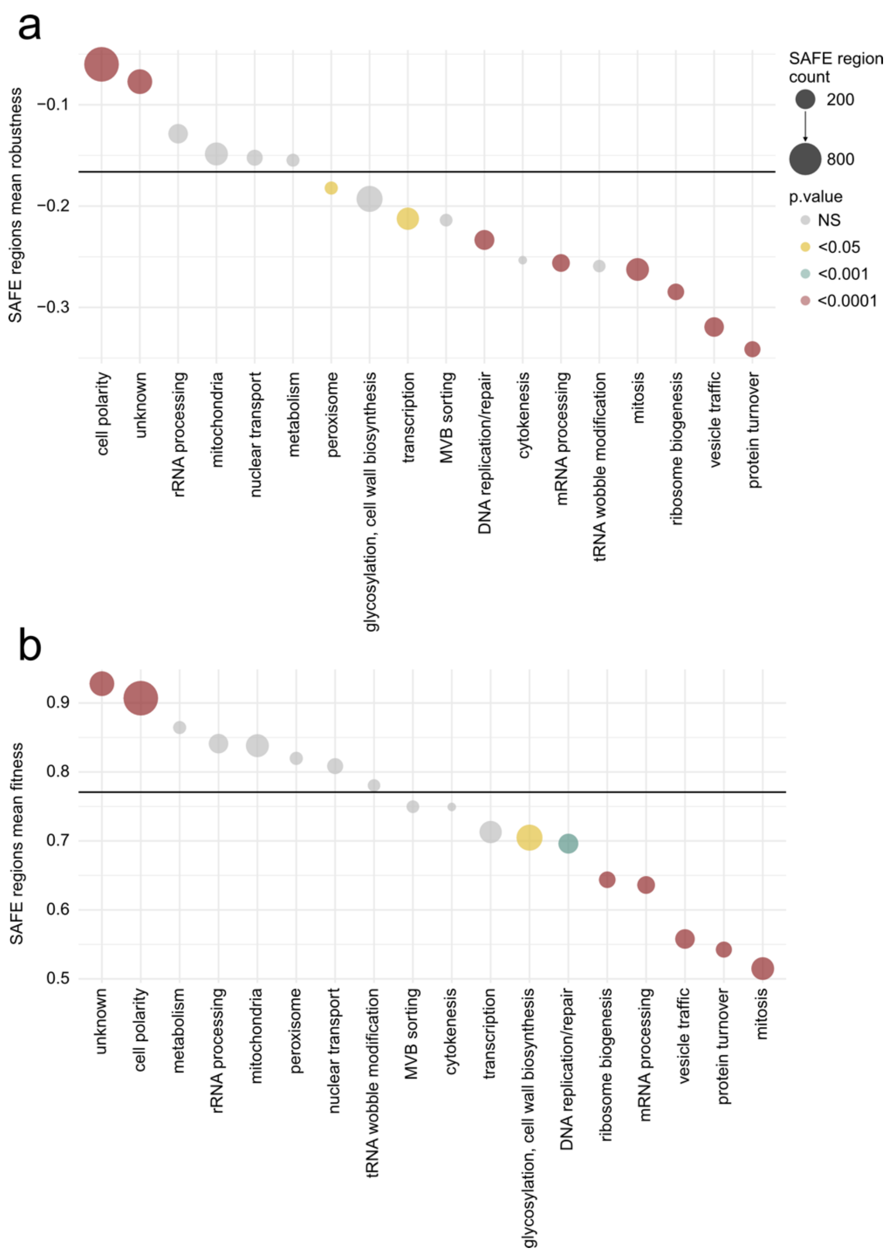

Figure S2. SAFE regions mean fitness and robustness (reference dataset). *a) The x-axis represents the SAFE regions from the reference dataset, ordered based on the mean robustness plotted on the y-axis. The horizontal black line corresponds to the mean robustness across all mutants from the reference dataset. Each dot's size correlates with the count of mutants whose deleted genes are associated with the respective region on the x-axis, ranging from 25 for "cytokinesis" to 964 for "cell polarity". Dot colors represent p-values derived from Wilcoxon tests comparing each group (x-axis) with the entire reference dataset. b) replicates the structure of panel a) but focuses on mean fitness instead of robustness.*

Figure S3.

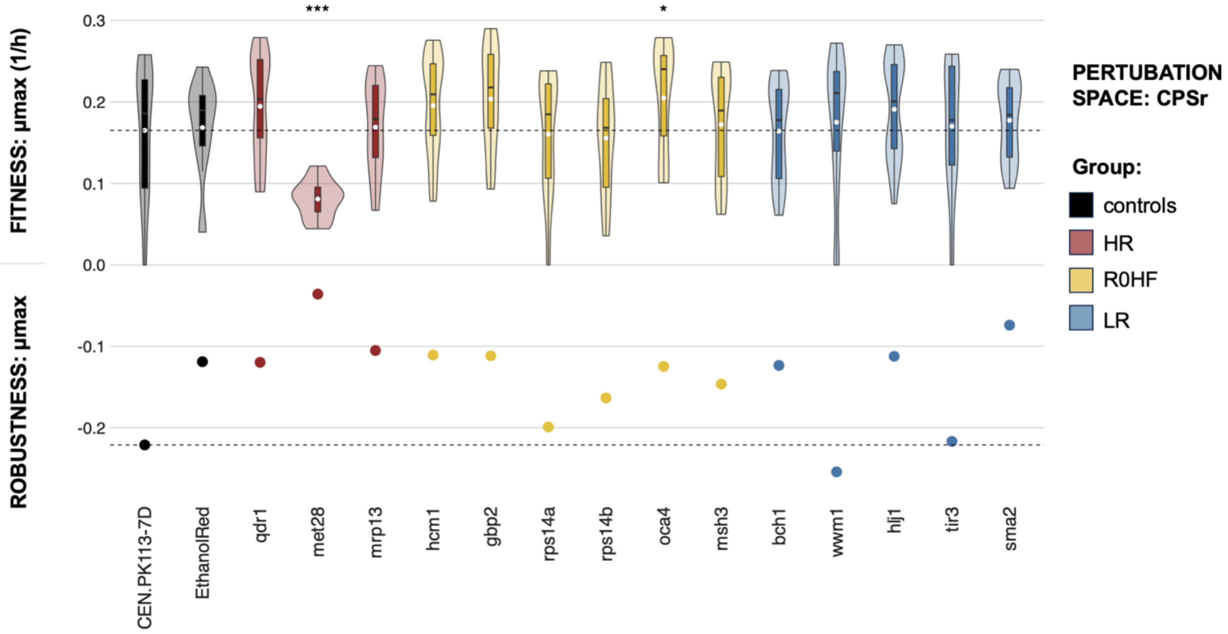

Figure S3. Fitness and robustness in the CPSr (Delft medium + antifungal agents used in the reference dataset). *The y-axis (0;0.3) represents the maximum specific growth rate (1/h) across all strains and perturbations (n=24), with the black line in the boxplot indicating the median of the distribution and the white dot indicating the mean. Significant difference between the parental strain and the mutants is denoted by p-values obtained from Wilcox tests (*p<0.05, ****p<0.0001, not significant scores are omitted). Robustness of the maximum growth rate is shown by dots on the y-axis (-0.2;0), while the horizontal dashed black line marks the robustness and fitness of the CEN.PK113-7D parental strain. Groups are colored differently based on analysis of the reference dataset (HR: best robustness score different from zero; R0HF: robustness equal to zero and best fitness score; LR: worst robustness score; controls: CEN.PK113-7D parental and Ethanol Red).*

Figure S4.

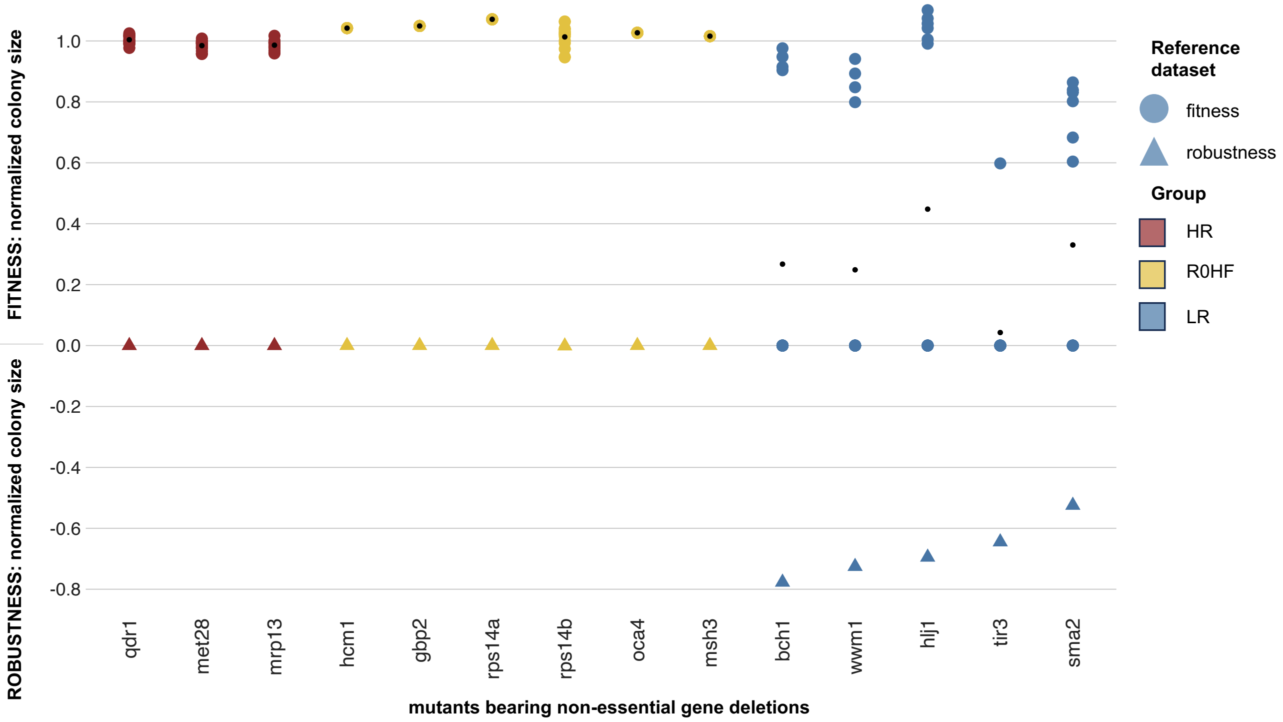

Figure S4. Fitness and robustness in the CPSo. *Fitness values for the normalized colony size are plotted on the y-axis (0;1) with circles (n=14) for each mutant bearing a non-essential gene deletion (x-axis). The small black dot represents the mean across the 14 conditions tested in the reference dataset for each mutant. Robustness of each mutant is plotted as a triangle (-0.8;0). Groups are colored differently based on analysis of the reference dataset (HR: best robustness score different from zero; R0HF: robustness equal to zero and best fitness score; LR: worst robustness score).*
